# Structural basis for p53 binding to its nucleosomal target DNA sequence

**DOI:** 10.1093/pnasnexus/pgac177

**Published:** 2022-09-04

**Authors:** Masahiro Nishimura, Yoshimasa Takizawa, Kayo Nozawa, Hitoshi Kurumizaka

**Affiliations:** Laboratory of Chromatin Structure and Function, Institute for Quantitative Biosciences, The University of Tokyo, 1-1-1 Yayoi, Bunkyo-ku, Tokyo 113-0032, Japan; Department of Biological Sciences, Graduate School of Science, The University of Tokyo, 1-1-1 Yayoi, Bunkyo-ku, Tokyo 113-0032, Japan; Laboratory of Chromatin Structure and Function, Institute for Quantitative Biosciences, The University of Tokyo, 1-1-1 Yayoi, Bunkyo-ku, Tokyo 113-0032, Japan; Laboratory of Chromatin Structure and Function, Institute for Quantitative Biosciences, The University of Tokyo, 1-1-1 Yayoi, Bunkyo-ku, Tokyo 113-0032, Japan; School of Life Science and Technology, Tokyo Institute of Technology, 4259 Nagatsuta-cho, Midori-ku, Yokohama 226-8501, Japan; Laboratory of Chromatin Structure and Function, Institute for Quantitative Biosciences, The University of Tokyo, 1-1-1 Yayoi, Bunkyo-ku, Tokyo 113-0032, Japan; Department of Biological Sciences, Graduate School of Science, The University of Tokyo, 1-1-1 Yayoi, Bunkyo-ku, Tokyo 113-0032, Japan

**Keywords:** p53, nucleosome, pioneer transcription factor, cryo-EM

## Abstract

The tumor suppressor p53 functions as a pioneer transcription factor that binds a nucleosomal target DNA sequence. However, the mechanism by which p53 binds to its target DNA in the nucleosome remains elusive. Here we report the cryo-electron microscopy structures of the p53 DNA-binding domain and the full-length p53 protein complexed with a nucleosome containing the 20 base-pair target DNA sequence of p53 (p53BS). In the p53-nucleosome structures, the p53 DNA-binding domain forms a tetramer and specifically binds to the p53BS DNA, located near the entry/exit region of the nucleosome. The nucleosomal position of the p53BS DNA is within the genomic p21 promoter region. The p53 binding peels the DNA from the histone surface, and drastically changes the DNA path around the p53BS on the nucleosome. The C-terminal domain of p53 also binds to the DNA around the center and linker DNA regions of the nucleosome, as revealed by hydroxyl radical footprinting. These results provide important structural information for understanding the mechanism by which p53 binds the nucleosome and changes the chromatin structure for gene activation.

Significance Statement In response to DNA damage, the transcription factor p53 stimulates the expression of various genes involving tumor suppressors by binding to the p53 binding DNA sequence, which is frequently embedded within the nucleosome. Here, we present cryo-EM structures of the p53-nucleosome complexes. In the structures, p53 peels the nucleosomal DNA region containing the p53 binding DNA sequence from the histone core, and specifically binds the DNA as a tetramer. Biochemical analyses revealed that the flexible p53 C-terminal domain, which contains an additional DNA-binding domain, binds around the center and linker DNAs of the nucleosome. These results provide novel insights for understanding the mechanism by which p53, as a pioneer transcription factor, recognizes its binding sequence in chromatin.

## Introduction

**Fig. 1.**
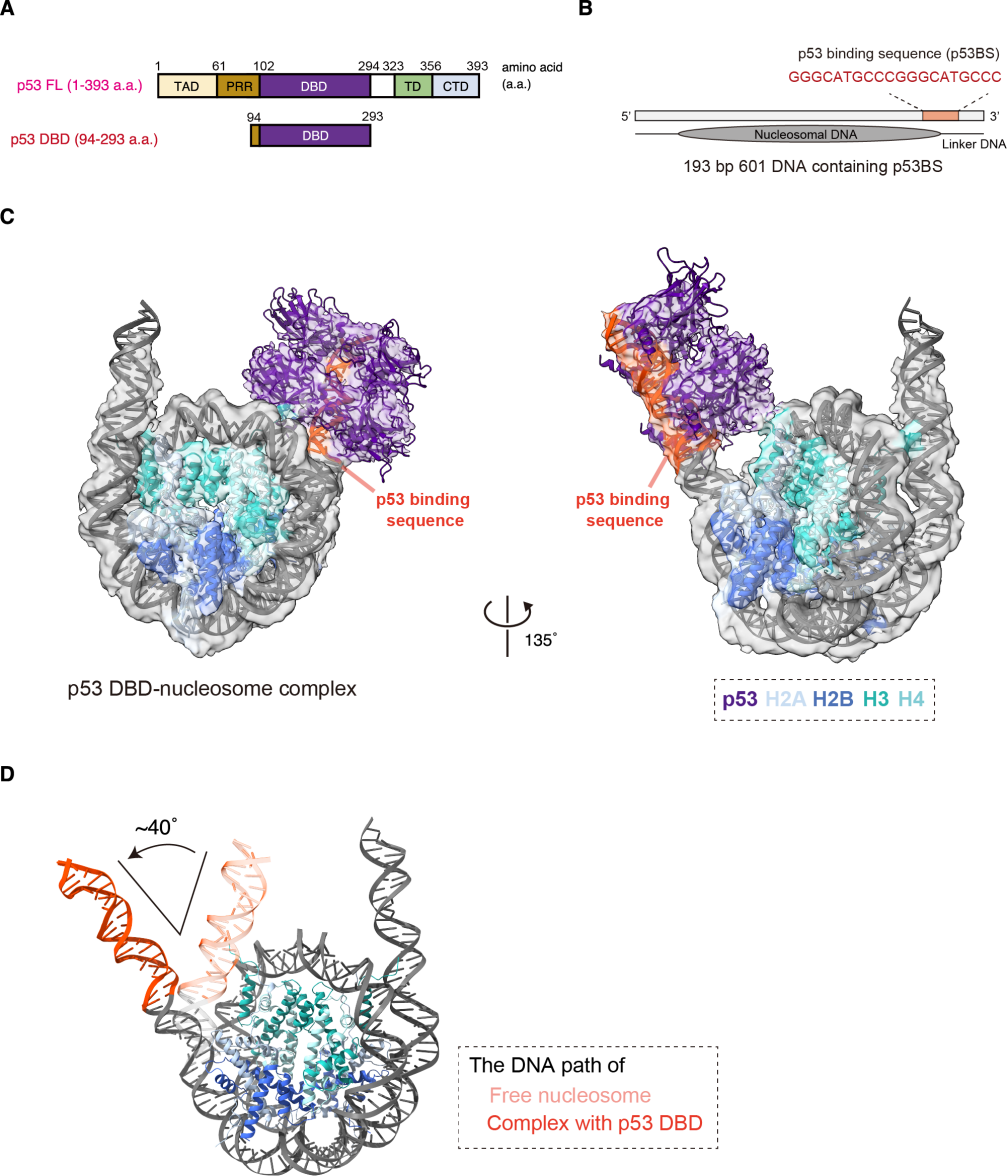
p53 binding induces nucleosomal linker DNA release from the histone octamer. (A) Schematic representations of the full-length p53 (p53 FL) and p53 DBD. The transcription activation domain 1/2 (TAD1/2), proline-rich region (PRR), core DNA-binding domain (DBD), tetramerization domain (TD), and C-terminus domain (CTD) are indicated. (B) Schematic illustration of the nucleosomal DNA construct containing the p53 binding sequence (193 bp 601 DNA). The p53BS DNA region, which directly contacts the histone surface in the absence of p53, is highlighted. (C) Cryo-EM structure of the p53 DBD-nucleosome complex. The atomic model of the p53 DBD-nucleosome complex is fitted into the transparent cryo-EM map. The p53BS DNA is shown in orange. (D) Structural comparison between the p53 DBD-nucleosome complex and the nucleosome containing the same DNA sequence. Schematic representations of the linker DNA paths of the 193 bp nucleosome with and without p53 DBD binding. The atomic model of the cryo-EM structure of the nucleosome containing the 193 bp 601 DNA was superimposed on the nucleosome structure in the p53 DBD-nucleosome complex. The nucleosomes are aligned by the histone octamer, and the p53BS DNA is shown in orange. The angle of the DNA distortion was measured by Pymol.

The p53 protein is the product of a tumor suppressor gene, and functions as a transcription factor that induces the expression of genes related to cell cycle, apoptosis, senescence, and DNA repair (1). The human p53 protein is composed of 393 amino acid residues, which form the transcription activation domain 1 (TAD1, residues 1 to 40), the transcription activation domain 2 (TAD2, residues 41 to 60), the proline-rich region (PRR, residues 61 to 93), the DNA-binding domain (DBD, residues 102 to 293), the tetramerization domain (TD, residues 323 to 353), and the C-terminal domain (CTD, residues 364 to 393) (Fig. 1A) (2). The DBD region plays an important role in DNA binding by p53, especially for its specific interaction with the target DNA sequence, termed the p53 binding sequence (p53BS). Mutations in p53 have been found in about 50% of cancer patients, and most of the mutations are accumulated within the DBD (3, 4). These findings suggest that defects in the sequence-specific DNA binding of p53 may be a major cause of tumorigenesis.

In eukaryotes, genomic DNA is highly compacted as chromatin. The basic chromatin unit is a nucleosome, in which approximately 150 base pairs (bps) of DNA are bound to a histone octamer containing two each of histones H2A, H2B, H3, and H4 (5). The genomic DNA region wrapped in the nucleosome becomes inaccessible to DNA-binding proteins, such as transcription factors (6, 7). In fact, most transcription factors cannot bind their target DNA sites located within the nucleosome (8). In contrast, a group of transcription factors, termed pioneer transcription factors, somehow specifically bind their target DNA sequences in the nucleosome, and promote transcription activation in chromatin (9).

The p53 tumor suppressor is a pioneer transcription factor (10–12), and binds to the nucleosomal p53BS DNA when it is located near the nucleosomal edge (13–15). Previous crystallographic studies revealed that the p53 DBD specifically binds to the naked p53BS DNA as a tetramer (16, 17). Another DNA-binding region is also present in the p53 CTD region, which is intrinsically disordered and confers the sequence-independent DNA-binding activity (18). However, the mechanism by which p53 binds the nucleosome containing the p53BS has remained elusive.

In the present study, we reconstituted the nucleosome containing the p53BS DNA in the position found in the native nucleosome at the p21 promoter region, and determined the cryo-electron microscopy (cryo-EM) structures of the p53-nucleosome complexes. Deletion analyses of p53 domains coupled with hydroxyl radical footprinting revealed the nucleosomal DNA binding by the p53 DBD and CTD regions. These results provide structural information for understanding how p53 binds to the nucleosomal target DNA sequence in chromatin.

## Results

### Cryo-EM structures of the p53-nucleosome complex

We reconstituted the nucleosome with a 193 bp DNA containing a modified 601 sequence for the structural analysis with p53 (Fig. 1B). The reconstituted nucleosome contained linker DNAs on both sides, and the 20 bp p53BS DNA is inserted at the border of the entry/exit and linker DNA regions on one side of the nucleosome. Consequently, about half of the p53BS DNA directly contacts the nucleosomal histones. This p53BS DNA location in the nucleosome is reportedly found in the p21 promoter region (13), and may be a native nucleosomal position of the p53 binding sites in the genome (14). For structural analysis, we first employed the p53 DBD (Fig. 1A). We purified the p53 DBD peptide (residues 94 to 293) as a recombinant protein, and incubated it with the reconstituted nucleosome. The resulting p53 DBD-nucleosome complex was prepared by ultracentrifugation with sucrose and paraformaldehyde gradients (GraFix) (Fig. S1). From the 4,502 cryo-EM images obtained by the Krios G4 microscope (Thermo Fisher Scientific), equipped with a K3 BioQuantum direct electron detector (Gatan), 2,452,876 particles were collected and analyzed with the Relion3.1 software (19) (Figs. S2 and S3, and Table S1).

Density corresponding to the p53 DBD was observed in approximately 70% of particles, but most of the p53 DBD map was not obvious because of the flexibility and/or multiple conformations of the p53 DBD bound to the nucleosomal DNA (Fig. S2A). We then determined the cryo-EM structure of the p53 DBD-nucleosome complex with particles, in which the p53 DBD density was clearly visible (Figs. 1C and S2A). The p53 DBD reportedly binds to its target p53BS DNA as a tetrameric form (16, 17). In the complex, the p53 DBD tetramer was clearly visualized at the periphery of the nucleosome, near the linker DNA with the p53BS DNA. The 20 bp p53BS DNA is composed of a tandem repeat of the palindromic 5’ GGGCA TGCCC 3’ 10-mer, and each p53 DBD protomer specifically recognizes a 5’ GGGCA 3’ sequence (16, 17). To accomplish the specific DNA binding in the nucleosome, the p53 DBD tetramer peels 15 bp of the nucleosomal DNA from the histone surface at the entry/exit region, and the p53 DBD binds the p53BS DNA site. Consequently, the linker DNA path of the p53BS is bent by about 40 degrees upon p53 binding (Fig. 1D).

To determine whether the full-length p53 (p53 FL) targets the nucleosomal p53BS DNA by the same mechanism as the p53 DBD, we purified the p53 FL protein and prepared the p53 FL-nucleosome complex (Figs. S4 to S6). In this experiment, we employed a 169 bp DNA sequence, which lacks one linker DNA region without the p53BS DNA, to avoid the non-specific DNA binding of p53 FL to the linker DNA. Interestingly, the p53 DBD region, which specifically binds to the entry/exit region of the nucleosomal DNA, was clearly visualized as a tetramer, although the p53 N-terminal region (TAD1, TAD2, and PRR), and C-terminal region (TD and CTD) were not observed in the cryo-EM maps, probably due to their flexible nature and/or multiple conformations (Fig. S7A). Similar to the p53 DBD, p53 FL also peels the DNA from the histone surface, and binds the p53BS DNA located at the entry/exit region of the nucleosome (Fig. S7B).

**Fig.2.**
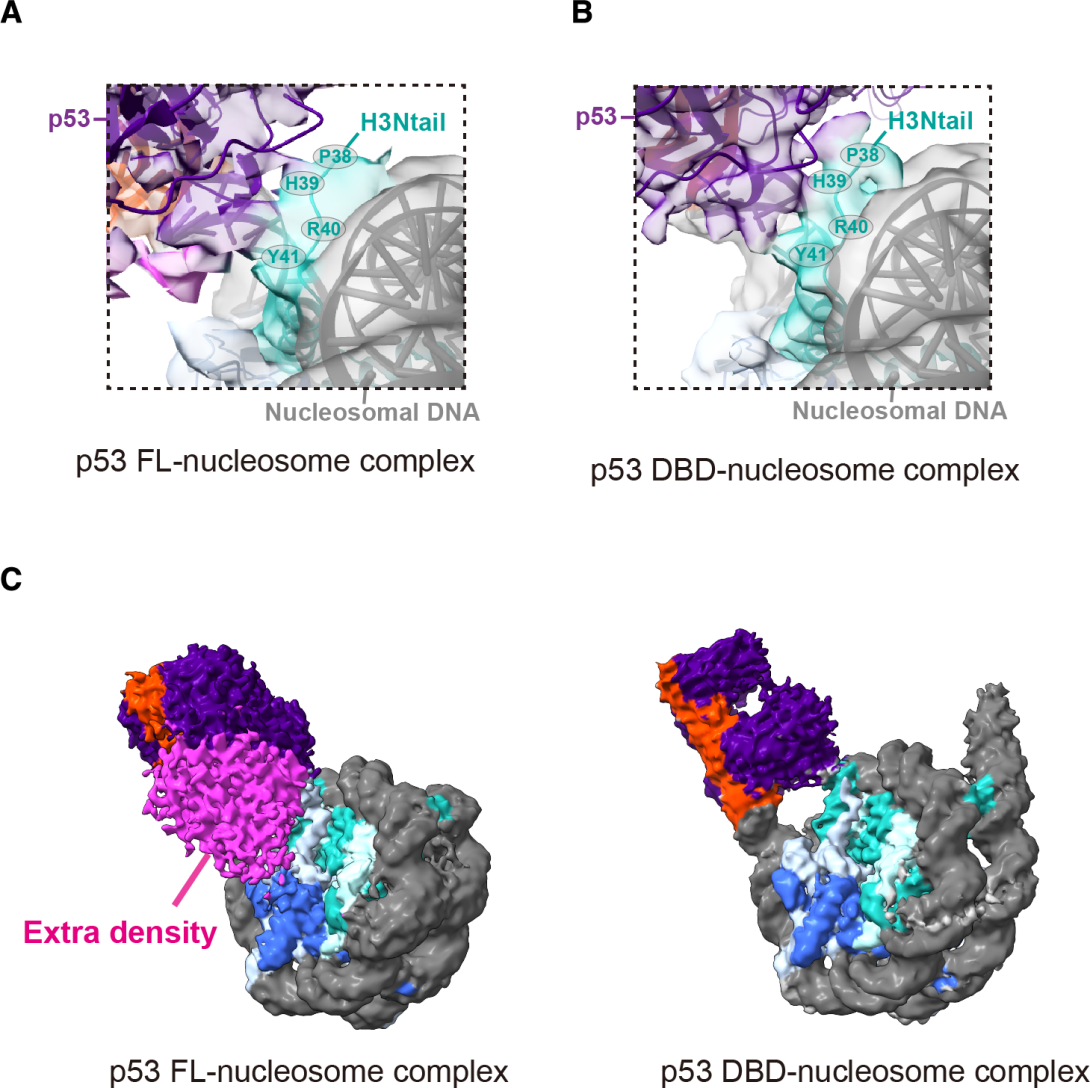
Structural comparison between the full-length p53-nucleosome and the p53 DBD-nucleosome complexes. (A) Close-up view of the interface between the p53 FL and the H3 N-terminal tail (H3Ntail) in the p53 FL-nucleosome complex. For the visualization of the H3Ntail, the map of the p53 FL-nucleosome complex is shown in the lower contour level (0.0105). The H3 amino acid residues near the interface are shown by the one-letter code. (B) Close-up view of the interface between the p53 DBD and the H3Ntail in the p53 DBD-nucleosome complex. For the visualization of the H3Ntail, the map of the p53 DBD-nucleosome complex is shown in the lower contour level (0.0094). The H3 amino acid residues near the interface are shown by the one-letter code. (C) The cryo-EM maps of the p53 FL-nucleosome (left panel) and the p53 DBD-nucleosome (right panel) complexes. The extra density observed in the p53 FL-nucleosome complex map is shown in pink.

In both the p53 FL-nucleosome and p53 DBD-nucleosome structures, the p53 DBD may contact the nucleosomal histone H3 tail near the His39 residue (Fig. 2A and B). These p53-nucleosomal histone interactions could enhance the specific binding of p53 to the nucleosome. In addition, a comparison of the structures of the p53 FL-nucleosome complex and the p53 DBD-nucleosome complex revealed extra p53 density (Fig. 2C), which may correspond to the N- and/or C-terminal region of p53.

### p53 binding to nucleosomal DNA

**Fig.3.**
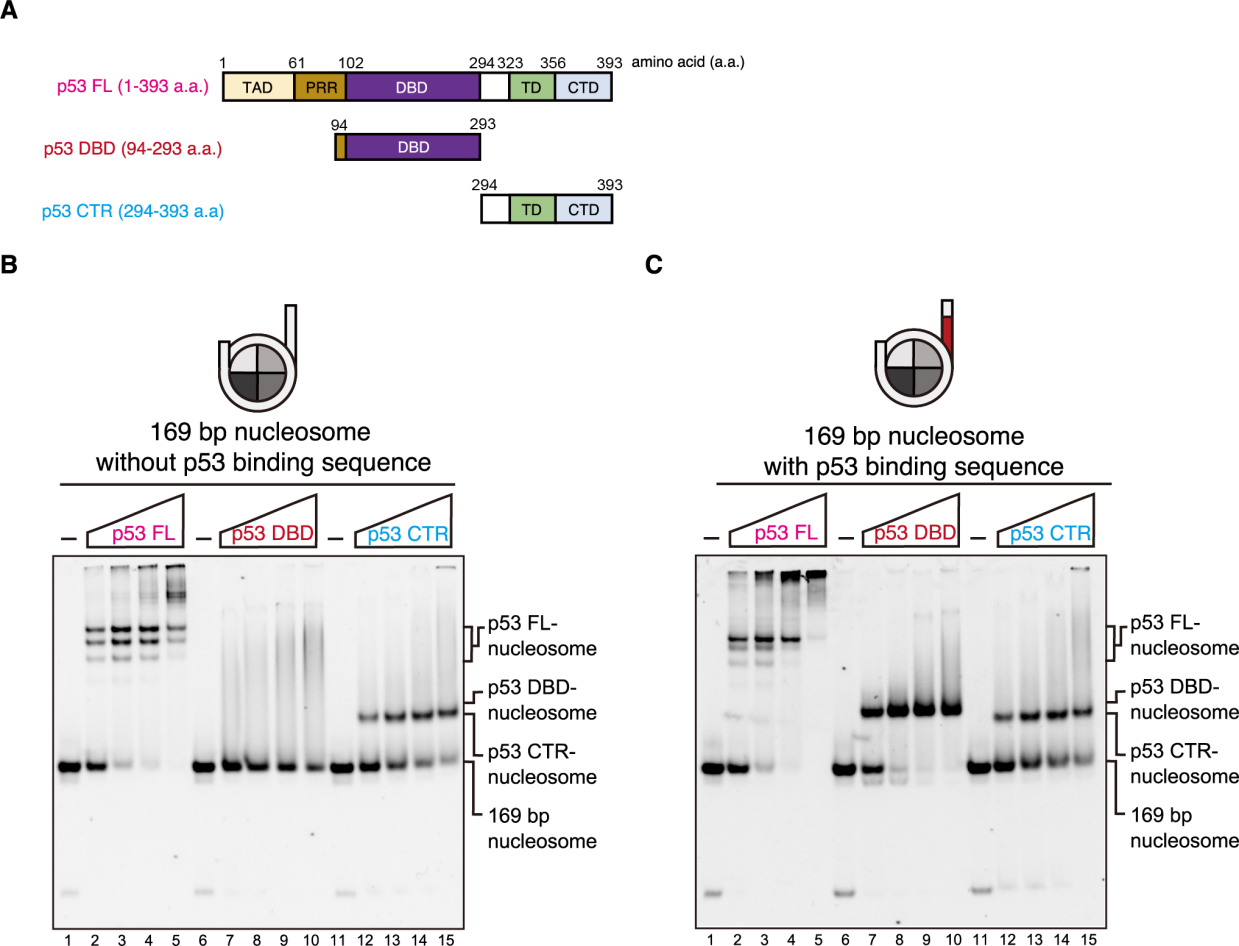
Nucleosome binding by the p53 domains. (A) Schematic representations of the p53 FL, p53 DBD, and p53 CTR. The transcription activation domain 1/2 (TAD1/2), proline-rich region (PRR), core DNA-binding domain (DBD), tetramerization domain (TD), and C-terminus domain (CTD) are indicated. (B) Electrophoretic mobility shift assay for p53 binding to the nucleosome without the p53 binding sequence. Nucleosome (0.1 µM) was mixed with p53 FL (lanes 1 to 5), p53 DBD (lanes 6 to 10), or p53 CTR (lanes 11 to 15), and analyzed by non-denaturing polyacrylamide gel electrophoresis. The protein concentrations are 0, 0.2, 0.4, 0.6, and 0.8 µM. (C) Electrophoretic mobility shift assay for p53 binding to the nucleosome with the p53 binding sequence. Nucleosome (0.1 µM) was mixed with p53 FL (lanes 1 to 5), p53 DBD (lanes 6 to 10), or p53 CTR (lanes 11 to 15), and analyzed by non-denaturing polyacrylamide gel electrophoresis. The protein concentrations are 0, 0.2, 0.4, 0.6, and 0.8 µM.

To further dissect the p53 domain contribution to the nucleosome binding, we prepared a p53 mutant, p53 CTR (residues 294 to 393) containing another DNA-binding region of the p53 CTD (Figs. 3A and S8). On the one hand, consistent with previous reports, the p53 FL protein bound the nucleosomes with and without the p53BS DNA (Fig. 3B and C, lanes 1 to 5) (14, 15). On the other hand, the p53 DBD alone hardly bound to the nucleosome without the p53BS DNA, but formed a specific complex with the nucleosome containing the p53BS DNA (Fig. 3B and C, lanes 6 to 10). Consistently, the p53 CTR peptide binds to both nucleosomes with and without the p53BS DNA (Fig. 3B and C, lanes 11 to 15), suggesting that the sequence-independent nucleosome binding is mediated by the p53 CTD.

**Fig.4.**
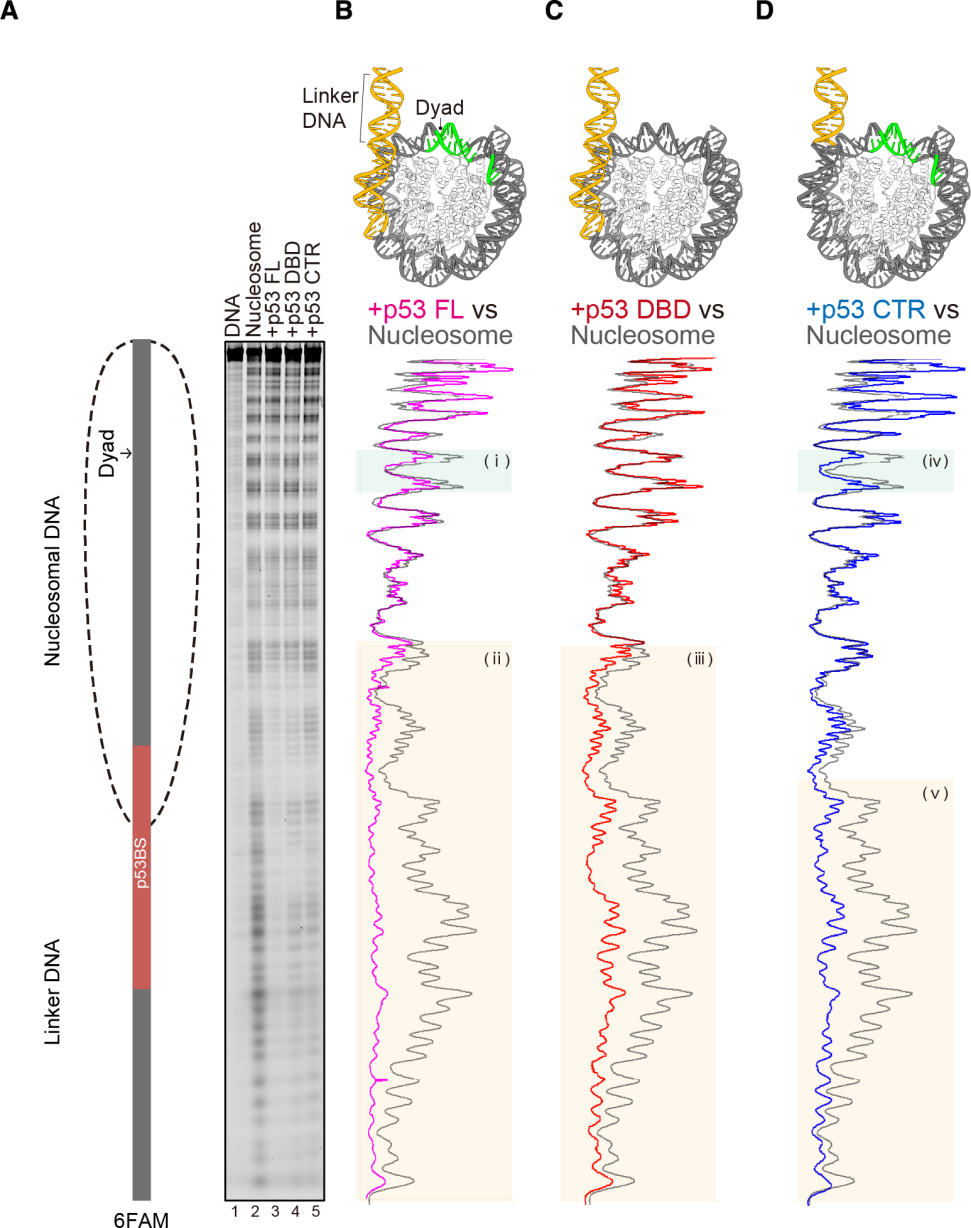
The p53-DNA interaction in the nucleosome. (A) Hydroxyl-radical footprinting of the p53-nucleosome complexes. The 6FAM fluorescence is conjugated on the nucleosomal linker DNA end. Lanes 1 and 2 indicate the control experiments with the 169 bp 601 DNA and the 169 bp nucleosome with the p53BS, respectively. Lanes 3, 4, and 5 indicate the experiments with p53 FL, p53 DBD, and p53 CTR, respectively. (B) A densitometry plot corresponding to the experiment with p53 FL (purple), compared to that corresponding to the nucleosome without p53 (gray). (C) A densitometry plot corresponding to the experiment with p53 DBD (red), compared to that corresponding to the nucleosome without p53 (gray). (D) A densitometry plot corresponding to the experiment with p53 CTR (blue), compared to that corresponding to the nucleosome without p53 (gray). The DNA regions around the nucleosomal dyad (sites i and iv) and the linker DNA (sites ii, iii, and v) that are protected by p53 binding are highlighted by pale green and beige boxes, respectively. Structural representations of the DNA regions protected by p53 FL, p53 DBD, and p53 CTR are shown above each densitometry plot in panels (B, C, and D). The protected DNA regions are highlighted by green and yellow colors.

To clarify the interactions of the p53 regions with the nucleosomal DNA, we performed a hydroxyl radical footprinting analysis (Figs. 4 and S9). The hydroxyl radical attacks solvent-accessible DNA strands, and cleaves the DNA backbone at the attack sites (20). Therefore, the DNA strand facing the protein-binding surface would be protected from cleavage by the hydroxyl radical. Consequently, the DNA wrapped in the nucleosome exhibited the characteristic cleavage pattern with about a 10 bp periodicity, in which the DNA regions facing the nucleosomal histones are periodically protected from the hydroxyl radical attack (Fig. 4A, lanes 1 and 2). Consistent with the cryo-EM structure, the p53BS DNA and its proximal linker DNA region are substantially protected from the hydroxyl radical attack in the presence of p53 FL, indicating that p53 specifically binds to the p53BS DNA in the nucleosome (Fig. 4B).

In addition to the p53BS DNA region, the hydroxyl radical hypersensitive sites around the nucleosomal dyad are also protected by the p53 FL binding (Fig. 4B). Intriguingly, the p53 DBD alone did not protect the dyad DNA, although it protected the p53BS DNA and linker DNA region (Fig. 4C). This suggests that, in the nucleosome, p53 binding to the dyad DNA region is probably mediated by the p53 CTD. Consistent with this idea, the p53 CTR peptide clearly protected the dyad DNA region, as well as the linker DNA (Fig. 4D). Therefore, we conclude that the dyad DNA binding by p53 may be facilitated by the p53 CTD binding.

## Discussion

In the present study, we determined the cryo-EM structures of the p53-nucleosome complexes, in which the p53 DBD specifically binds to its target p53BS DNA sequence located in the natural nucleosomal position at the entry/exit region. In the structures, the p53 DBD tetramer peels the DNA from the histone surface, thus drastically changing the orientation of the linker DNA containing the p53BS DNA. Since the linker DNA orientation is a key determinant for the higher-order chromatin architecture, p53 binding to the nucleosomal entry/exit site could change the chromatin structure around its binding sites. Consistent with this idea, p53 binding to its target p53BS DNA reportedly induced changes in the local chromatin environments in cells (12). The p53-mediated changes of the chromatin environment may be coupled with histone modifications and chromatin remodeling (21). Therefore, the alteration of the nucleosomal linker DNA path generated by the p53-specific DNA binding may be a prerequisite for gene activation by recruiting downstream factors, such as histone modifiers and chromatin remodelers.

The nucleosomal linker DNA re-orientation observed in the p53-bound state could be induced with the p53 DBD lacking the N- and C-terminal regions, indicating that the p53 DBD alone is sufficient for the specific DNA binding in the nucleosome. Intriguingly, the p53 mutants and splicing variants lacking the C-terminal DNA-binding region are proficient in the activation of p53-mediated genes (22, 23). These facts suggest that the changes of the chromatin structure and dynamics induced by the p53 DBD binding may play an essential role in the activation of p53-regulated genes. Of note, in their complexes with nucleosomes, several transactivating factors such as the chromatin remodeler Chd1 (24), BAF (25, 26), and the Oct4-Sox2 pioneer transcription factors (27) also exhibit similar nucleosomal linker DNA re-orientations. Therefore, the linker DNA re-orientation may be a common mechanism for promoting transcription activation by a certain class of nucleosome binding factors.

The present study also demonstrated that the C-terminal DNA binding region of p53 is involved in its nucleosome binding. Mice lacking the p53 CTD reportedly exhibit enhanced senescence and apoptosis (28). The splicing isoforms, p53β and p53γ, lacking the p53 CTD are strongly expressed in cancer cells (29, 30). These facts imply that the p53 CTD is important for the maintenance of cellular homeostasis. We found that the p53 CTD binds the nucleosomal DNA around the dyad and linker DNA regions, and its nucleosome binding may occur in a DNA sequence-independent manner (14). In contrast, the p53 DBD alone scarcely binds to the nucleosome without the p53BS DNA sequence (Fig. 3). Therefore, the p53 CTD may play a role in non-specific nucleosome binding in cells. A large excess of p53 relative to the number of its target p53BS DNAs is frequently found in certain types of cells (31), in which most of the p53 may non-specifically bind to nucleosomes. Nucleosome binding by the p53 CTD without DNA sequence specificity may also contribute to establishing proper chromatin structures by its dyad and linker DNA binding activities. Further studies will be required to understand the function of the non-specific nucleosome binding mediated by the p53 CTD in cells.

## Materials and Methods

### Histone purification

The human histones were bacterially produced and purified by the method described previously (32). Each DNA sequence encoding H2A (Uniprot ID: P04908), H2B (Uniprot ID: P06899), H3.1 (Uniprot ID: P68431), and H4 (Uniprot ID: P62805) was cloned into the pET-15b vector (Novagen) with an N-terminal His-tag connected by a thrombin cleavage site. The proteins were purified with Ni-NTA agarose beads (QIAGEN), and the His-tag was removed by thrombin protease digestion. The untagged proteins were purified by cation exchange chromatography on a Mono S column (Cytiva) and stored as freeze-dried powders at 4°C.

### Histone octamer reconstitution

The histone octamer was reconstituted and purified by the method previously described (32). The purified histone H2A, H2B, H3.1, and H4 powders were mixed at an equal molar ratio in denaturing buffer, containing 50 mm Tris-HCl (pH 7.5), 7 m guanidine hydrochloride, and 20 mm ß-mercaptoethanol. The histone octamer was then refolded by dialysis against refolding buffer, containing 10 mm Tris-HCl (pH 7.5), 2 m NaCl, 1 mm EDTA, and 5 mm ß-mercaptoethanol, and purified by size exclusion chromatography on a HiLoad 16/60 Superdex 200 prep grade column (Cytiva). The eluted fractions were flash-frozen and stored at −80°C.

### Nucleosome reconstitution

The nucleosomes were reconstituted by the salt dialysis method, as previously described (32). The DNA fragments for the nucleosome reconstitutions were amplified by PCR and purified by non-denaturing PAGE, using a Model 491 Prep Cell apparatus (Bio-Rad). Each DNA fragment was mixed with the purified histone octamer, and then dialyzed against reconstitution buffer-high, containing 10 mm Tris-HCl (pH 7.5), 2 m KCl, 1 mm EDTA (pH 8.0), and 1 mm DTT. Nucleosomes were reconstituted by decreasing the KCl concentration to that in reconstitution buffer-low, containing 10 mm Tris-HCl (pH 7.5), 0.25 m KCl, 1 mm EDTA (pH 8.0), and 1 mm DTT. The nucleosomes were then purified by non-denaturing polyacrylamide gel electrophoresis using a Model 491 Prep Cell apparatus (Bio-Rad), and stored at −80°C in nucleosome-buffer, containing 20 mm Tris-HCl (pH 7.5), 1 mm DTT, and 5% glycerol.

### Purification of p53

The p53 FL, p53 DBD, and p53 CTR proteins were bacterially produced and purified as previously described (15). The DNA sequence encoding human p53 (Uniprot ID: Q761V2) was cloned into the pGEX-6P-1 vector (Cytiva) with an N-terminal GST-tag connected by a PreScission cleavage site. The p53 production was induced with 0.5 mm IPTG. The proteins were purified with GS4B beads (Cytiva), and the GST-tag was removed by PreScission protease digestion. The untagged p53 protein was further purified by column chromatography with Heparin Sepharose 6 Fast Flow affinity resin (Cytiva) and HiLoad16/60 Superdex 200 prep grade resin (Cytiva), in p53-buffer containing 30 mm Tris-HCl (pH 7.5), 500 mm NaCl, and 2 mm ß-mercaptoethanol.

### Electrophoretic mobility shift assay

The fluorescently labeled nucleosome (final concentration of 0.1 µm) was mixed with either 0, 0.4, 0.6, or 0.8 µm of the p53 protein, and incubated at 25°C for 30 min in buffer containing 20 mm Tris-HCl (pH 7.5 or pH 8.0), 100 mm NaCl, 0.4 mm ß-mercaptoethanol, 0.7 mm DTT, and 0.01% NP−40. The reaction products were then fractionated by 5% non-denaturing polyacrylamide gel electrophoresis in 0.5xTBE buffer, and visualized by ethidium bromide staining or Cy5 fluorescence signal detection, using an Amersham Typhoon imager (Cytiva).

### Preparation of the p53-nucleosome complexes for cryo-EM analysis

The p53-nucleosome complexes for cryo-EM analysis were prepared by the GraFix method (33). For the p53 DBD-nucleosome complex, the 193 bp nucleosome (final concentration, 4.14 µm) was mixed with the p53 DBD in a 6-fold molar ratio, in buffer containing 25 mm Tris-HCl (pH 7.5 or pH 8.0), 250 mm NaCl, 1 mm ß-mercaptoethanol, and 0.5 mm DTT, and incubated at 25°C for 30 min. The mixture was applied on the top of a 5% to 20% (w/v) sucrose gradient solution containing 10 mm HEPES-KOH pH 7.5, 20 mm NaCl, and 2 mm DTT, with an increasing concentration (0% to 2%) of paraformaldehyde (Electron Microscopy Sciences), and the samples were centrifuged at 27,000 rpm at 4°C for 16 hr, using an SW41 rotor (Beckman Coulter). After centrifugation, 400 µL aliquots of the sample were collected from the top of the gradient solution. The sample fractions were analyzed by non-denaturing polyacrylamide gel electrophoresis. The peak fractions were then collected and desalted with buffer, containing 20 mm HEPES-KOH (pH 8.0) and 2 mm TCEP (pH 8.0), using a PD-10 column (Cytiva). For the p53 FL-nucleosome complex, the 169 bp nucleosome (final concentration of 4 µm) was mixed with p53 FL in a 4-fold molar ratio, in buffer containing 22.5 mm Tris-HCl (pH 7.5 or pH 8.0), 225 mm NaCl, 0.9 mm ß-mercaptoethanol, 0.45 mm DTT, and 0.01% NP-40, and incubated at 25°C for 30 min. The p53 FL-nucleosome complex was then fixed and purified by the same method as described above.

### Cryo-EM grid preparation and data collection

For the cryo-EM specimen preparation, R1.2/1.3 200 mesh copper grids (Quantifoil) were washed with ethyl acetate and glow-discharged by soft plasma ion bombardment (PIB-10, Vacuum Device Inc.). Aliquots (2.5 µL) of the p53-nucleosome complex or the p53 DBD-nucleosome complex were applied to the Quantifoil grids in the Vitrobot Mark IV chamber (Thermo Fisher Scientific) at 100% humidity and 16°C, and then blotted and plunged into liquid ethane. Data collections were performed on a Krios G4 cryo-transmission electron microscope (Thermo Fisher Scientific) operated at 300 kV, using the EPU software. Digital micrographs of the p53-nucleosome complex and the p53 DBD-nucleosome complex were recorded on a K3 BioQuantum (Gatan) direct electron detector calibrated at a pixel size of 1.06 and 1.1 Å in the electron counting mode, using a slit width of 25 eV, and retaining 40 frames with total doses of 56.8 and 46 electron/Å^2^, respectively (Table S1).

### Image processing

The image processing was performed with the RELION3.1 software (34). All movie frames were aligned and dose-weighted using MOTIONCOR2 (35). The CTF estimation was performed by CTFFIND4 (36) from the digital micrographs. The particles were automatically picked with a box size of 280 × 280 pixels. Contaminated junk particles were removed through the processes of 2D classification and 3D classification. The 3D classification processes for the p53-nucleosome complexes were performed, followed by particle polishing and a few rounds of CTF refinement. The resolution of the refined 3D map was estimated by the gold standard Fourier Shell Correlation (FSC) with the 0.143 criterion (19). The local resolution maps and the angular distributions were calculated by RELION3.1.

### Model fitting and refinement

For the model building of the free nucleosome, the cryo-EM structure of the nucleosome containing a 145 bp Widom-601 sequence (PDB ID: 7OHC) (37) was placed into the corresponding cryo-EM map by rigid-body fitting in UCSF ChimeraX (38). The nucleosomal DNA edges were automatically fitted into the vacant volume in the cryo-EM map using ISOLDE (39). The linker DNA segments were then extended using COOT (40). The nucleotides and amino acid residues were manually corrected using COOT. Finally, the models were refined by the real-space refinement tool (41) in PHENIX (42) using base-pair and base-stacking restraints, and the restraints from the high-resolution crystal structure of the nucleosome (PDB ID: 5Y0C) (43) specified as the reference model.

For the p53-nucleosome complexes, the free nucleosome model and the crystal structure of the p53-DNA complex (PDB ID: 3KMD) (44) were placed into the corresponding cryo-EM map by rigid-body fitting in UCSF ChimeraX. The nucleosomal linker DNA portion was manually removed and the resulting DNA edge was automatically fitted into the vacant volume using ISOLDE (39). The DNA ends of the nucleosome and the p53-DNA complex were connected using COOT. The final models were validated by MolProbity (45) in PHENIX (42) (Table S1).

### Hydroxyl-radical footprinting assay

For the hydroxyl-radical footprinting experiments (46–48), the fluorescently labeled nucleosome (0.6 µm) was mixed with either p53 (2.4 µm), p53 DBD (3.6 µm), or p53 CTR (3.6 µm), and was incubated at 25ºC for 30 min. The buffer was exchanged into the reaction buffer, containing 5 mm Tris-HCl (pH 7.5), 5 mm NaCl, and 0.25 mm EDTA (pH 8.0), by four consecutive rounds of dilution and filtration using an Amicon Ultra-0.5 filter unit (MWCO 30 kDa). For the hydroxyl radical reaction, a 2.5 µL aliquot of 4 mm FeAmSO_4_/8 mm EDTA, 0.1 m sodium ascorbate, and 0.6% v/v H_2_O_2_ was simultaneously mixed with 50 µL of the sample, and then incubated for 2 min at room temperature. The reactions were terminated by adding a 5 μL aliquot of 100 mm thiourea and a 10 μL aliquot of 3 m sodium acetate (pH 5.2). The resulting DNA samples were purified by deproteinization and phenol/chloroform/isoamyl alcohol extraction, followed by ethanol precipitation. The DNA samples were denatured by Hi-Di^™^ formamide (Thermo Fisher Scientific) and separated by 8% Urea-PAGE in 1×TBE buffer. The Cy5 and 6FAM signals were detected with the Amersham Typhoon scanner (Cytiva). The gel images were analyzed with the ImageJ software (49).

## Supplementary Material

pgac177_Supplemental_File

## Data Availability

Coordinates and Cryo-EM maps have been deposited in the Electron Microscopy Data Bank and Protein Data Bank under accession codes EMD-33533, 7XZX (p53 DBD-nucleosome complex); EMD-33534, 7XZY (193 bp nucleosome); EMD-33535, 7XZZ (p53 FL-nucleosome complex); and EMD-33536, 7Y00 (169 bp nucleosome).
