# Supplementary material for: Structural basis for p53 binding to its nucleosomal target DNA sequence
Source: PNAS Nexus. 2022 Sep 4;1(4):pgac177. doi: 10.1093/pnasnexus/pgac177 (PMC9802185; doi:10.1093/pnasnexus/pgac177)
Supplement: pgac177_Supplemental_File [file pgac177_supplemental_file.pdf]

PNAS nexus

Supplementary Information for
Structural basis for p53 binding to its nucleosomal target DNA sequence

Masahiro Nishimura[1,2], Yoshimasa Takizawa[1], Kayo Nozawa[1,3], and Hitoshi Kurumizaka[1,2]

[1] Laboratory of Chromatin Structure and Function, Institute for Quantitative Biosciences, The University of Tokyo, 1-1-1 Yayoi, Bunkyo-ku, Tokyo 113-0032, Japan.
[2] Department of Biological Sciences, Graduate School of Science, The University of Tokyo, 1-1-1 Yayoi, Bunkyo-ku, Tokyo 113-0032, Japan.
[3] School of Life Science and Technology, Tokyo Institute of Technology, 4259 Nagatsuta-cho, Midori-ku, Yokohama 226-8501, Japan.

*Hitoshi Kurumizaka
Email: kurumizaka@iqb.u-tokyo.ac.jp

This PDF file includes:

Figures S1 to S9
Table S1

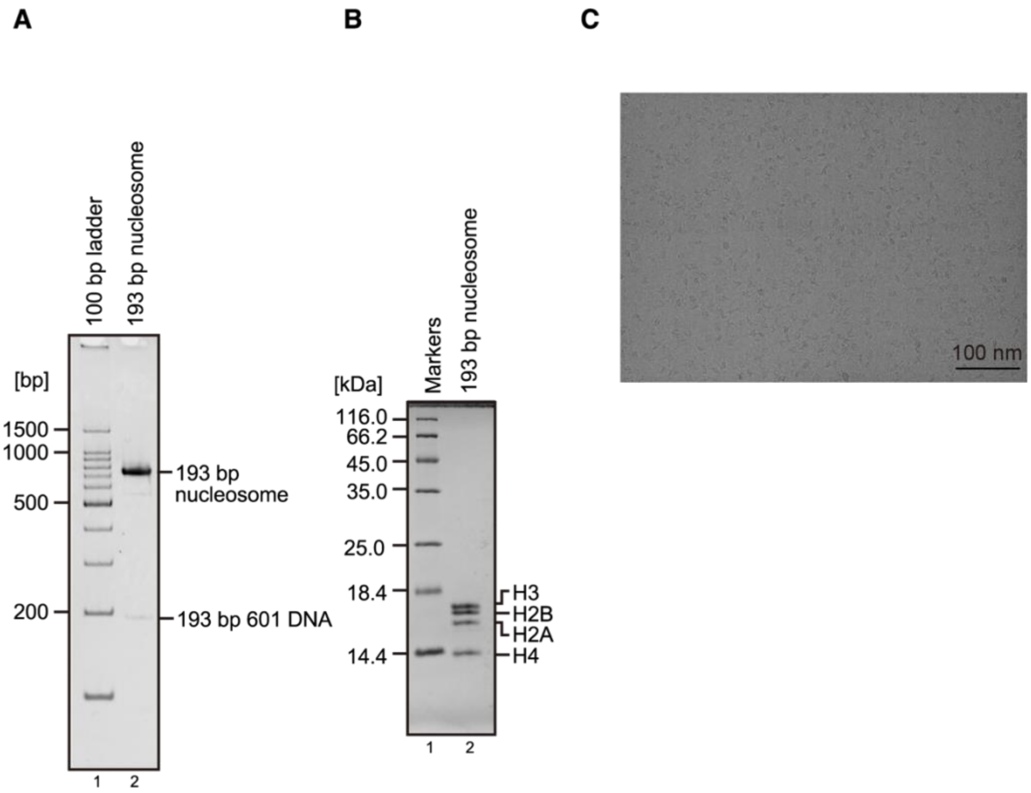

Fig. S1. Sample preparation for cryo-EM analysis of the p53 DBD-nucleosome complex.

(A) The nucleosome containing the 193 bp 601 DNA (193 bp nucleosome) was analyzed by non-denaturing gel electrophoresis with ethidium bromide staining. (B) The 193 bp nucleosome was analyzed by SDS-polyacrylamide gel electrophoresis with Coomassie Brilliant Blue staining. (C) A representative micrograph of the p53 DBD-nucleosome complex.

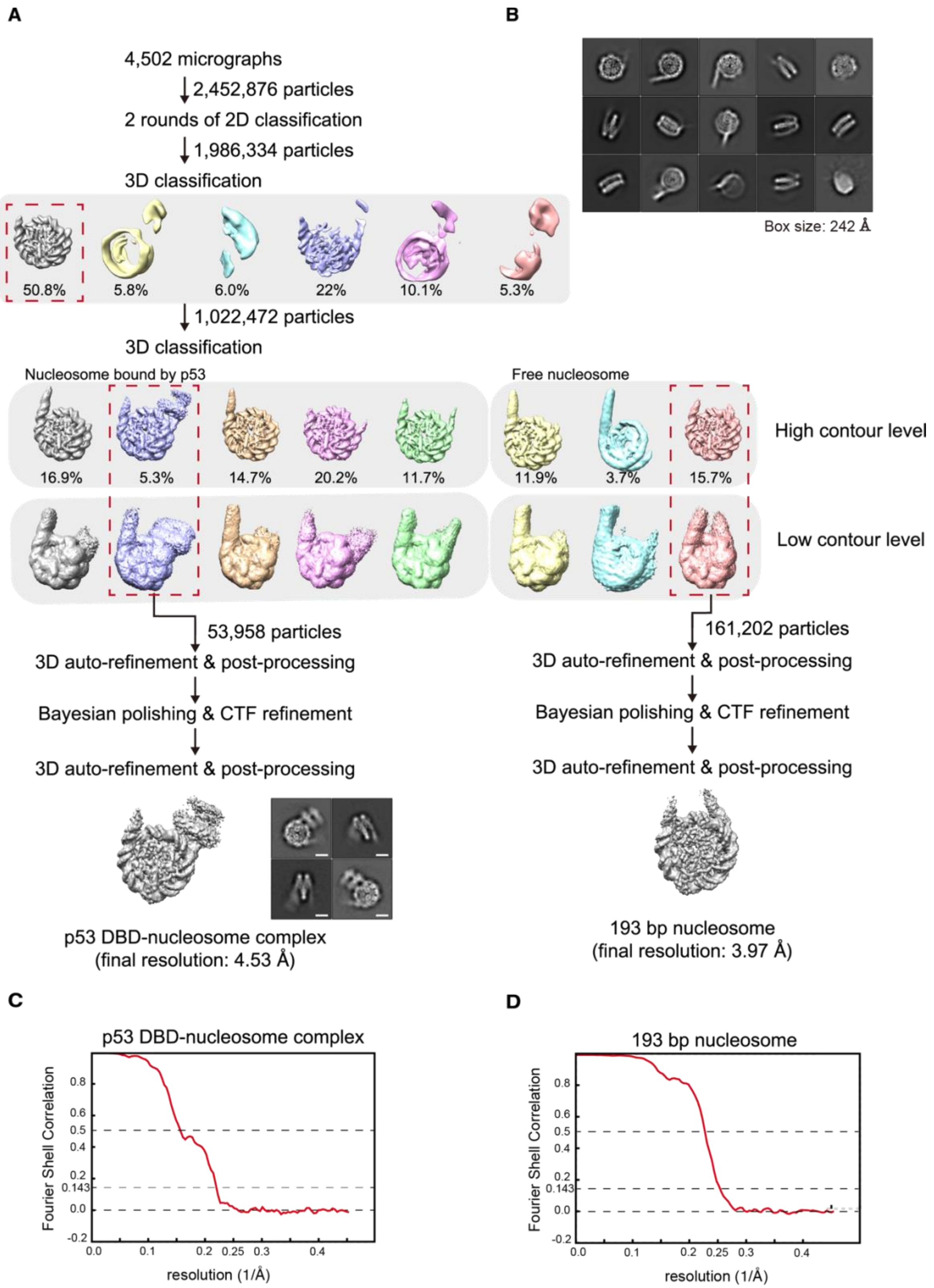
A
4,502 micrographs
2,452,876 particles
2 rounds of 2D classification
1,986,334 particles
3D classification
50.8%
5.8%
6.0%
22%
10.1%
5.3%
1,022,472 particles
3D classification
Nucleosome bound by p53
16.9%
5.3%
14.7%
20.2%
11.7%
Free nucleosome
11.9%
3.7%
15.7%
High contour level
Low contour level
53,958 particles
3D auto-refinement & post-processing
Bayesian polishing & CTF refinement
3D auto-refinement & post-processing
p53 DBD-nucleosome complex
(final resolution: 4.53 Å)
161,202 particles
3D auto-refinement & post-processing
Bayesian polishing & CTF refinement
3D auto-refinement & post-processing
193 bp nucleosome
(final resolution: 3.97 Å)
B
Box size: 242 Å
C
p53 DBD-nucleosome complex
Fourier Shell Correlation
resolution (1/Å)
D
193 bp nucleosome
Fourier Shell Correlation
resolution (1/Å)

Fig. S2. Cryo-EM structure determination of the p53 DBD-nucleosome complex.

(A) Flowchart of the single-particle analysis. Red dashed rectangles indicate the 3D classes selected for the subsequent processes. The 2D class average images using the final subsets of the particles are shown next to the reconstructed p53 DBD-nucleosome complex. The scale bars are 50 Å. (B) Representative 2D class average images with all particles. (C and D) Fourier shell correlations between half maps of the final reconstructions of the p53 DBD-nucleosome complex and the 193 bp nucleosome.

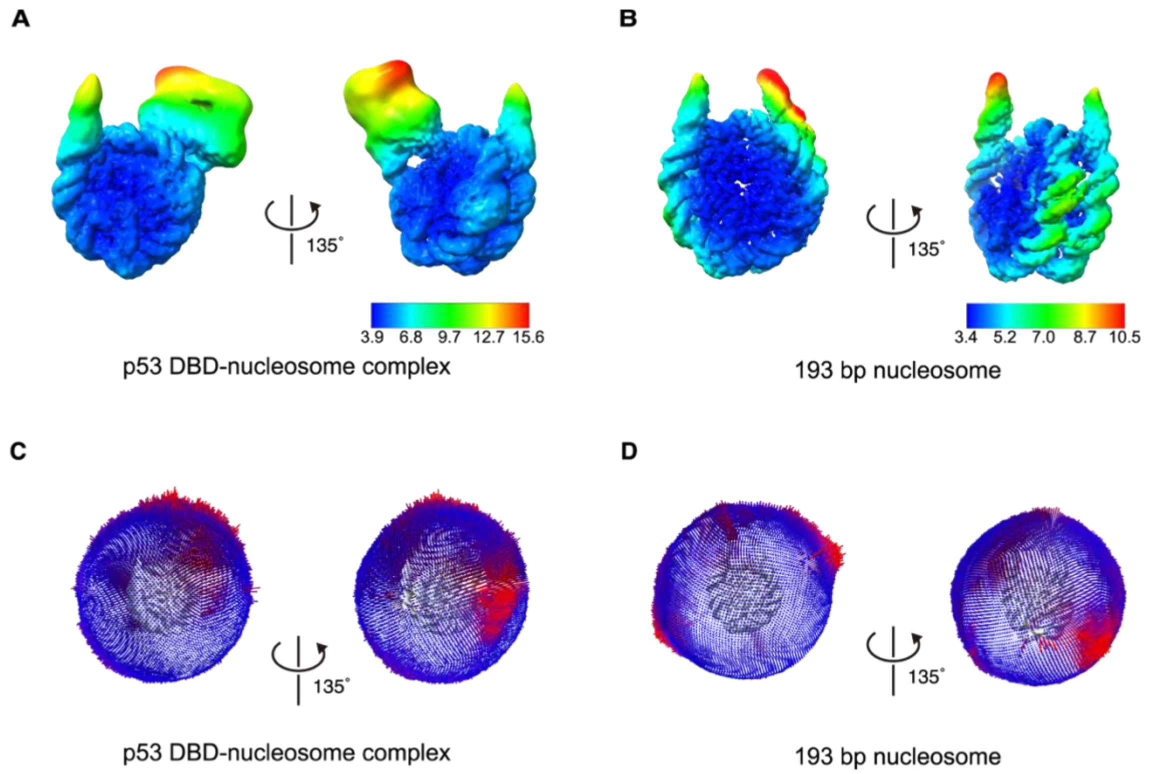

Fig. S3. Local resolutions and angular distributions, related to Fig. S2.

(A and B) Cryo-EM density surfaces of the p53 DBD-nucleosome complex and the 193 bp nucleosome, colored according to the local resolutions, respectively. (C and D) Angular distributions for particles in the final reconstructions of the p53 DBD-nucleosome complex and the 193 bp nucleosome, respectively.

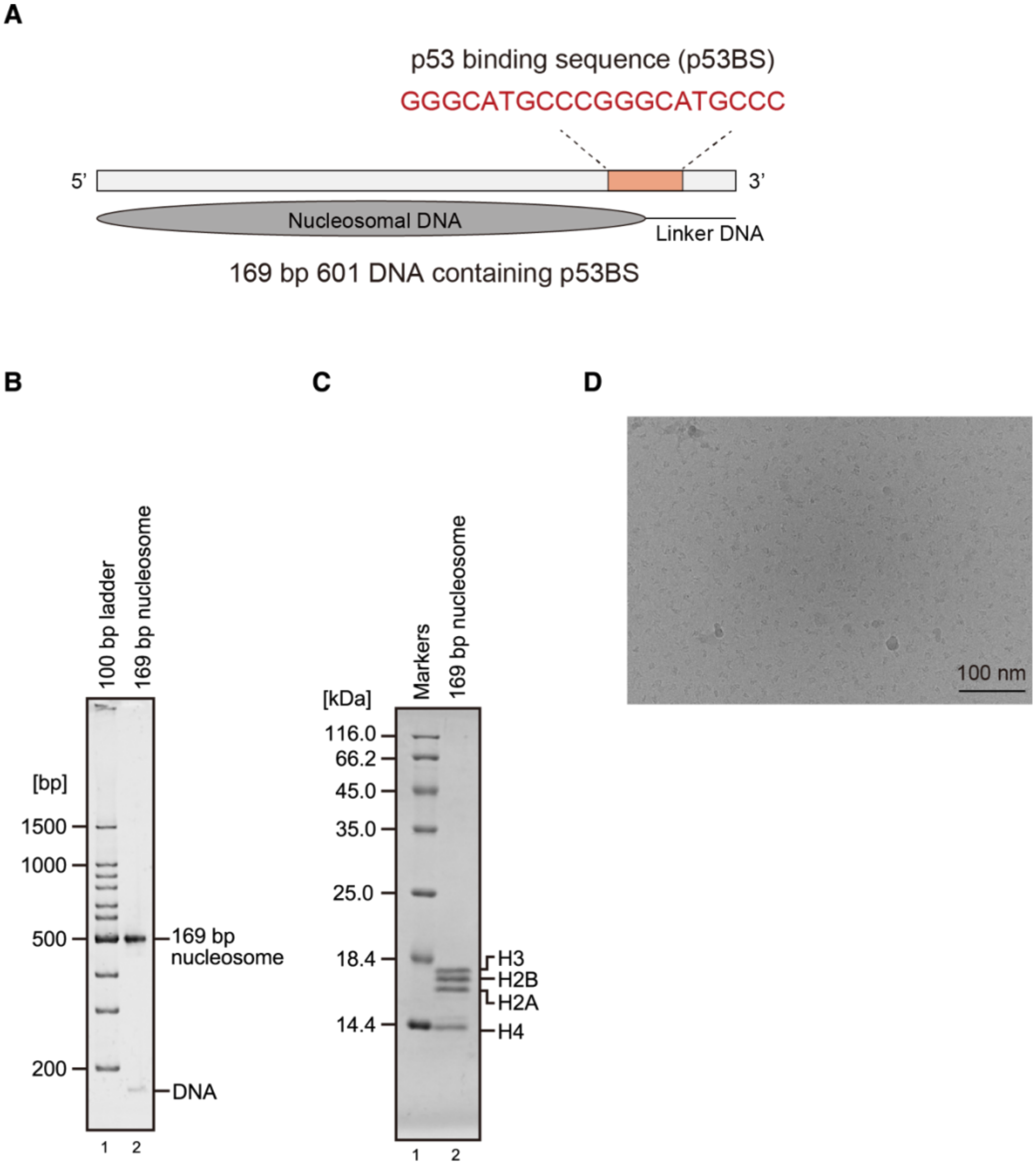

Fig. S4. Sample preparation for cryo-EM analysis of the p53 FL-nucleosome complex.

(A) Schematic illustration of the nucleosomal 169 bp DNA construct containing p53BS DNA (169 bp 601 DNA). (B) The 169 bp nucleosome was analyzed by non-denaturing gel electrophoresis with ethidium bromide staining. (C) The 169 bp nucleosome was analyzed by SDS-polyacrylamide gel electrophoresis with Coomassie Brilliant Blue staining. (D) A representative micrograph of the p53 FL-nucleosome complex.

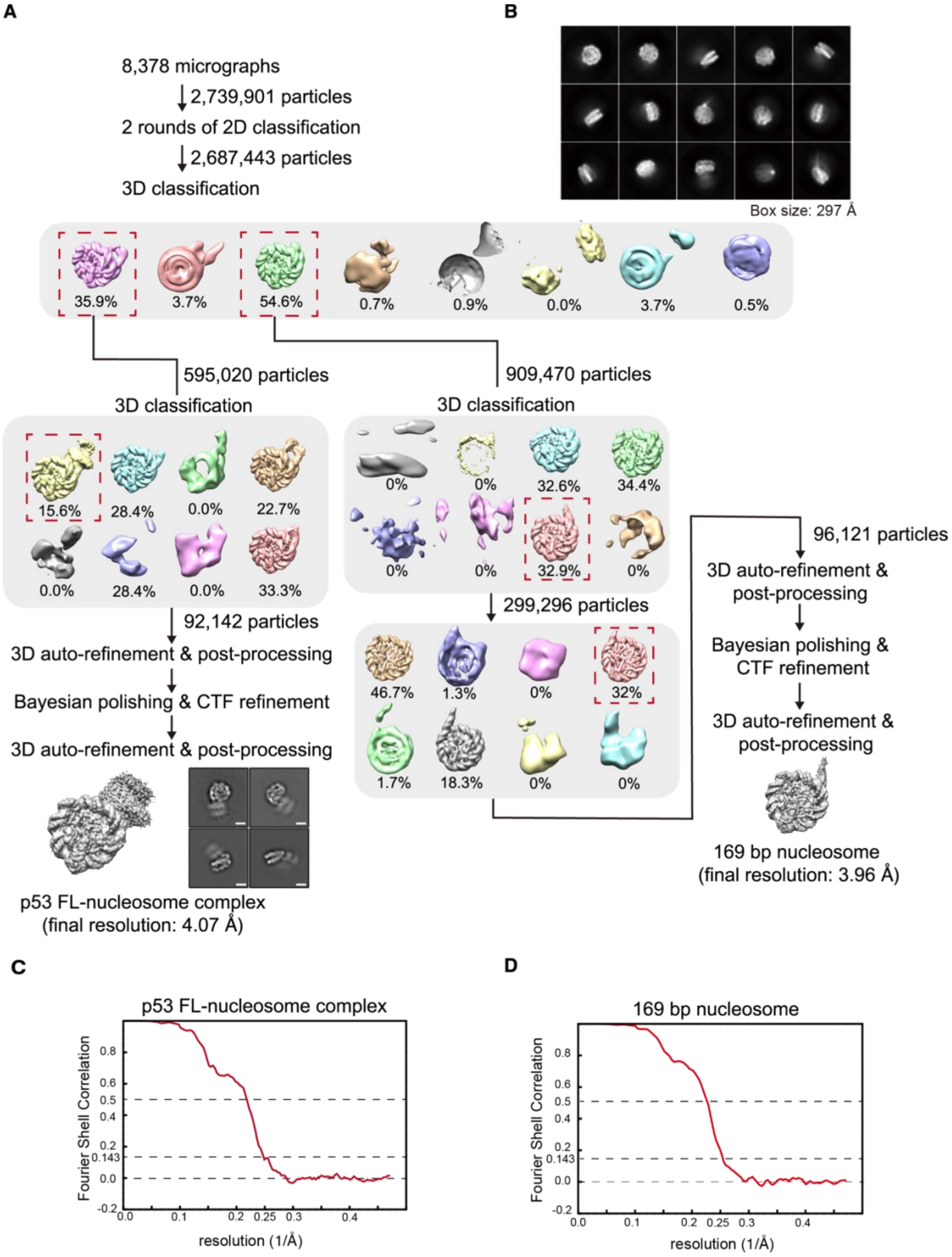

Fig. S5. Cryo-EM structure determination of the p53 FL-nucleosome complex.

(A) Flowchart of the single-particle analysis. Red dashed rectangles indicate the 3D classes selected for the subsequent processes. The 2D class average images using the final subsets of

the particles are shown next to the reconstructed p53 FL-nucleosome complex. The scale bars are 50 Å. (B) Representative 2D class average images with all particles. (C and D) Fourier shell correlations between half maps of the final reconstructions of the p53 FL-nucleosome complex and the 169 bp nucleosome.

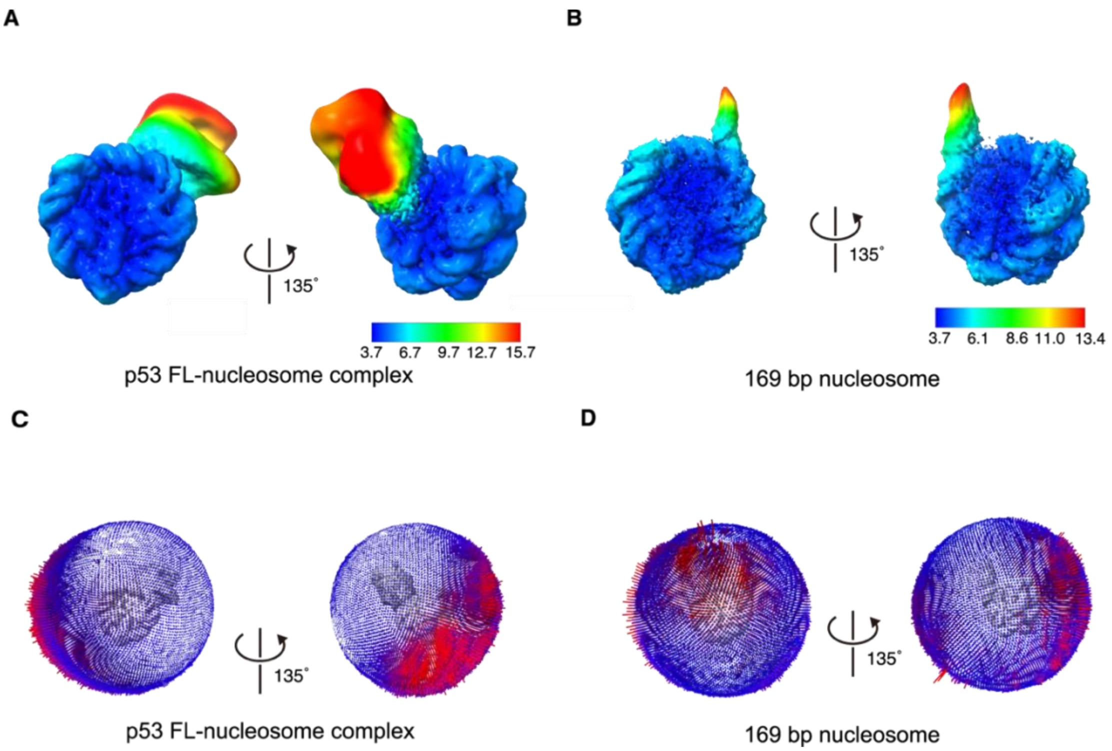

Fig. S6. Local resolutions and angular distributions, related to Fig. S5.

(A and B) Cryo-EM density surfaces of the p53 FL-nucleosome complex and the 169 bp nucleosome, respectively, colored according to the local resolutions. (C and D) Angular distributions for particles in the final reconstructions of the p53 FL-nucleosome complex and the 169 bp nucleosome.

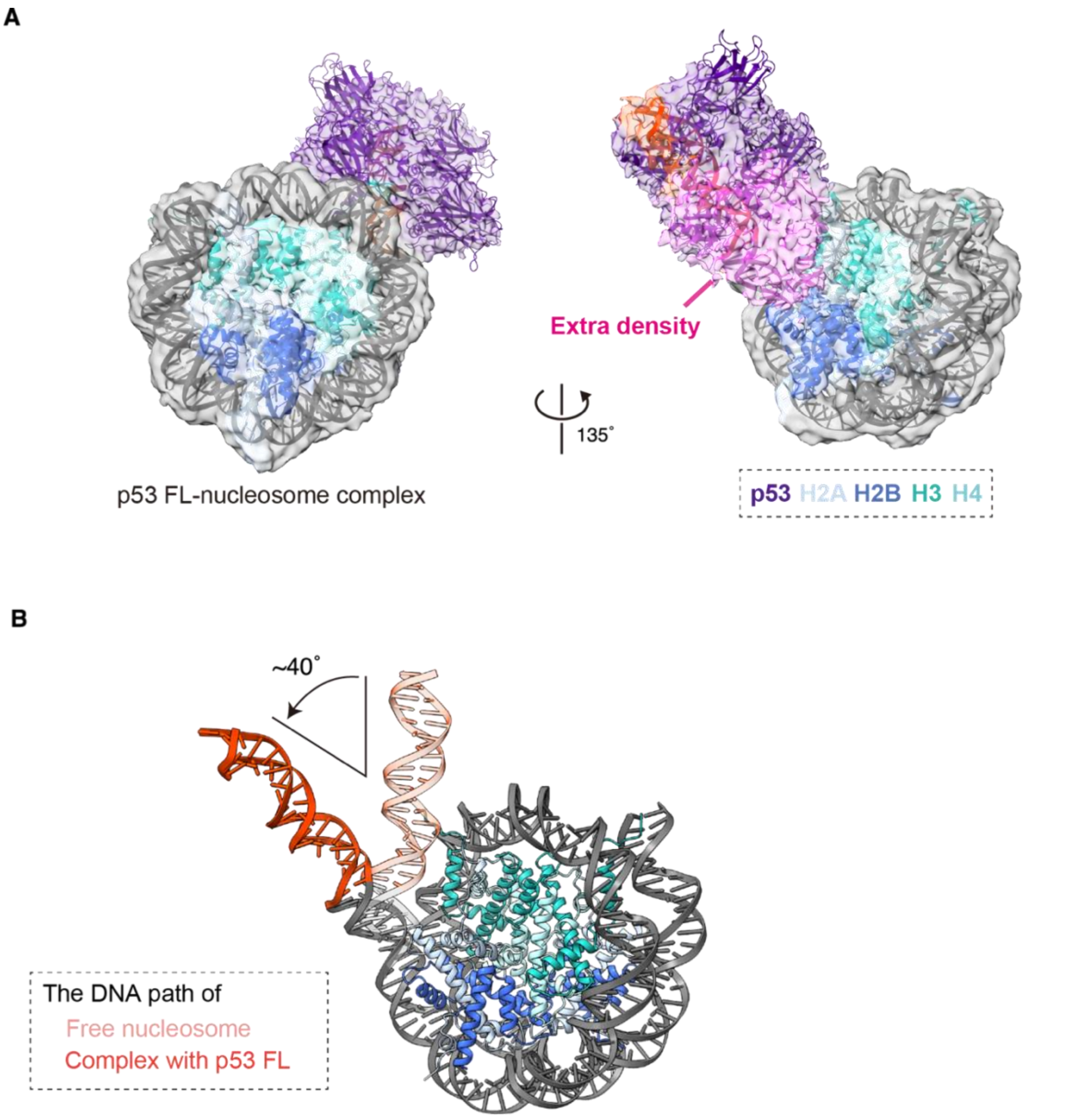

Fig. S7. Conformational change of the linker DNA region in the p53 FL-nucleosome.

(A) Cryo-EM structure of the p53 FL-nucleosome complex. The extra density within the p53 FL-nucleosome complex map is shown in pink, and the p53BS DNA is colored orange. (B) Schematic representation of the linker DNA paths of the 169 bp nucleosome with and without p53 FL binding. The atomic model of the cryo-EM structure of the nucleosome containing the 169 bp 601 DNA was superimposed on the nucleosome structure in the p53 FL-nucleosome complex. The nucleosomes are aligned by the histone octamer, and the p53BS DNA is shown in orange. The angle of the DNA distortion was measured by Pymol.

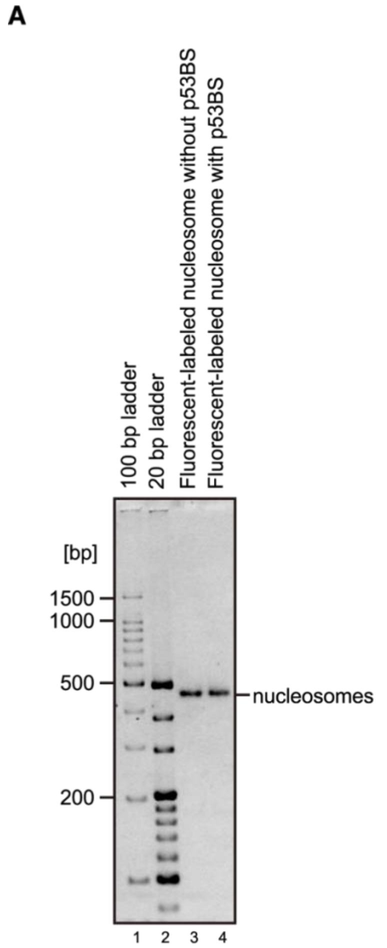

Fig. S8. Sample preparation for biochemical analyses.

(A) Nucleosomes containing the 169 bp 601 DNA with or without the p53BS DNA were reconstituted, purified, and analyzed by non-denaturing polyacrylamide gel electrophoresis. The nucleosomal DNA ends were fluorescently labeled with 6-FAM and Cy-5. The gel was stained with ethidium bromide. (B) The purified nucleosomes were analyzed by SDS-polyacrylamide gel electrophoresis with Coomassie Brilliant Blue staining. (C) Purified recombinant p53 FL, p53 DBD, and p53 CTR proteins were analyzed by SDS-polyacrylamide gel electrophoresis with Coomassie Brilliant Blue staining.

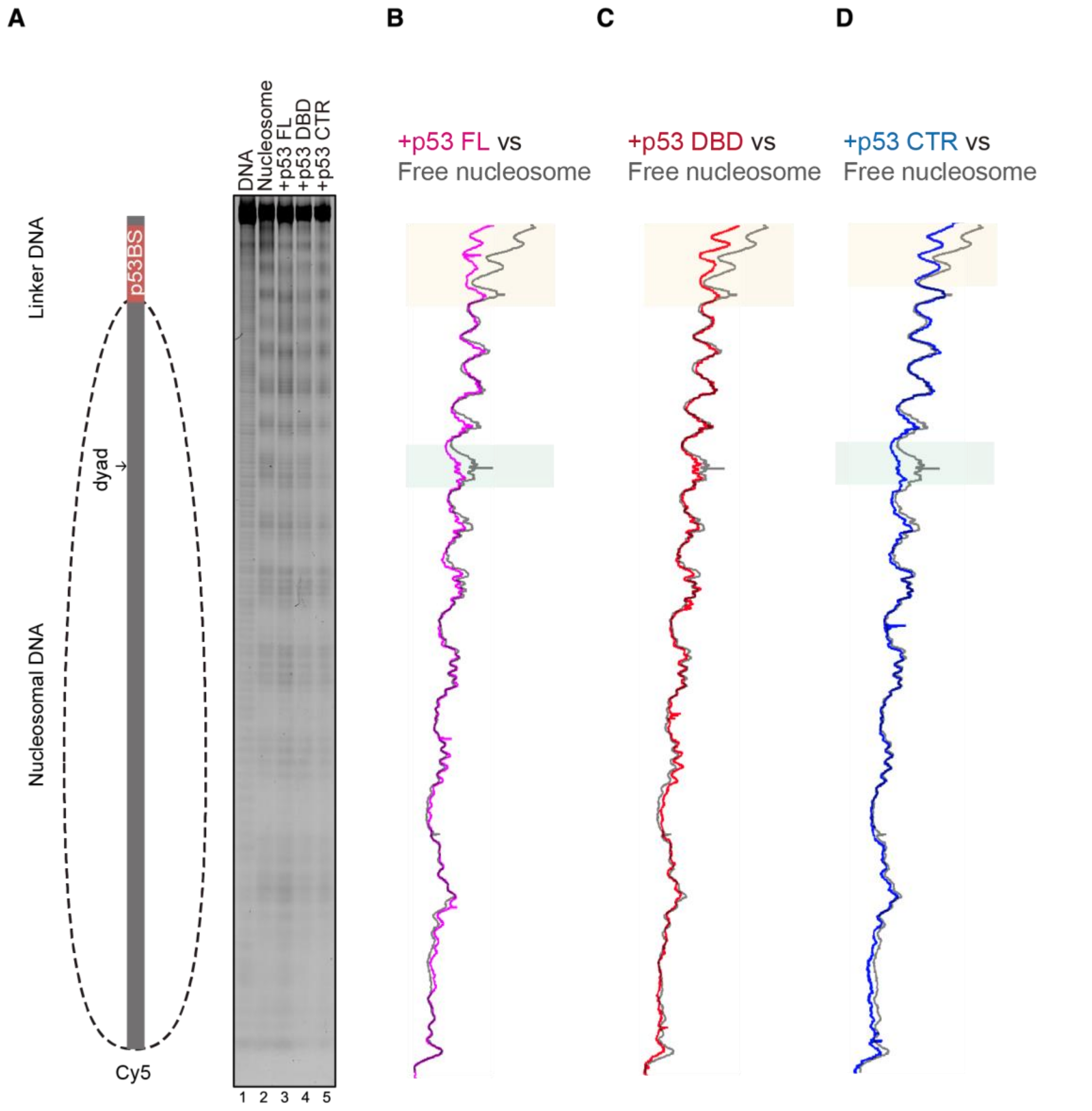

Fig. S9. Hydroxyl-radical footprinting analysis, related to Figure 4.

(A) Hydroxyl-radical footprinting analysis of the p53-nucleosome complexes, detected by the Cy5 signal. The Cy5 is conjugated on the opposite nucleosomal end relative to 6FAM, as shown in Figure 4. Lanes 1 and 2 indicate the control experiments with the 169 bp 601 DNA and the 169 bp nucleosome with the p53BS, respectively. Lanes 3, 4, and 5 indicate the experiments with p53 FL, p53 DBD, and p53 CTR, respectively. (B) A densitometry plot corresponding to the experiment with p53 FL (purple) is compared to that corresponding to the nucleosome without p53 (gray). (C) A densitometry plot corresponding to the experiment with p53 DBD (red) is compared to that corresponding to the nucleosome without p53 (gray). (D) A densitometry plot corresponding to the experiment with p53 CTR (blue) is compared to that corresponding to the nucleosome without p53 (gray). The DNA regions around the nucleosomal dyad and the linker DNA that are protected by p53 binding are highlighted by pale green and beige boxes, respectively.

Table S1. Cryo-EM data collection, refinement, and validation statistics.

	p53 DBD-nucleosome complex (EMDB-33533) (PDB ID 7XZX)	193 bp nucleosome (EMDB-33534) (PDB ID 7XZY)	p53 FL-nucleosome complex (EMDB-33535) (PDB ID 7XZZ)	169 bp nucleosome (EMDB-33536) (PDB ID 7Y00)
**Data collection and processing**				
Microscope	Krios G4			
Detector	K3 BioQuantum			
Magnification	81,000			
Voltage (kV)	300			
Electron exposure (e^-/Å^2)	46		56.8	
Defocus range (μm)	1 to 2.5		1 to 2.5	
Pixel size (Å)	1.1		1.06	
Symmetry imposed	C1			
Initial particle (no.)	2,452,876		2,739,901	
Final particle (no.)	53,958	161,202	92,142	96,121
Map resolution (Å)	4.56	3.967	4.065	3.957
FSC threshold	0.143	0.143	0.143	0.143
Map resolution range (Å)	3.9 to 15.6	3.4 to 10.5	3.7 to 15.7	3.7 to 13.4
**Refinement**				
Initial model used (PDB ID)	7OHC and 3KMD	7OHC	7OHC and 3KMD	7OHC
Map sharpening B factor (Å^2)	-15.96	-52.22	-126.63	-5.399
**Model composition**				
Protein residues	1554	762	1544	762
Nucleotide residues	342	330	310	310
**Validation**				
R.m.s. deviations				
Bond lengths (Å)	0.006	0.004	0.006	0.004

Bond angles (degree)	1.032	0.833	0.965	0.82
MolProbity score	1.55	1.67	1.56	1.62
Clashscore	10.75	14.66	11.08	12.97
Poor rotamers (%)	0.15	0.16	0.15	0.31
Ramachandran plot				
Favored (%)	98.3	98.12	98.3	98.53
Allowed (%)	1.7	1.88	1.7	1.47
Disallowed (%)	0	0	0	0
